# From concept to action: a united, holistic and One Health approach to respond to the climate change crisis

**DOI:** 10.1186/s40249-022-00941-9

**Published:** 2022-02-10

**Authors:** Renhe Zhang, Xu Tang, Jian Liu, Martin Visbeck, Huadong Guo, Virginia Murray, Catherine Mcgillycuddy, Bing Ke, Gretchen Kalonji, Panmao Zhai, Xiaoming Shi, Jiahai Lu, Xiaonong Zhou, Haidong Kan, Qunli Han, Qian Ye, Yong Luo, Jianmin Chen, Wenjia Cai, Huiling Ouyang, Riyanti Djalante, Alexander Baklanov, Lu Ren, Guy Brasseur, George Fu Gao, Lei Zhou

**Affiliations:** 1 Integrated Research on Disaster Risk International Centre of Excellence on Risk Interconnectivity and Governance on Weather/Climate Extremes Impact and Public Health & Institute of Atmospheric Sciences, Fudan University, Shanghai, 200438 China; 2 Science Division, United Nations Environment Programme (UNEP), United Nations Avenue, Gigiri, PO Box 30552, Nairobi, 00100 Kenya; 3 GEOMAR Helmholtz Centre for Ocean Research Kiel and Kiel University, Kiel, Germany; 4 Key Laboratory of Digital Earth Science, Aerospace Information Research Institute, Chinese Academy of Sciences, Beijing, China; 5 University of Chinese Academy of Sciences, Chinese Academy of Sciences, Beijing, China; 6 UK Health Security Agency, London, SE1 8UG UK; 7 The Administrative Center for China’s Agenda 21, Ministry of Science and Technology of China, Beijing, 100038 China; 8 Institute for Disaster Management and Reconstruction, Sichuan University, Chengdu, 610207 China; 9 State Key Laboratory of Severe Weather, Chinese Academy of Meteorological Sciences, Beijing, 100081 China; 10 China CDC Key Laboratory of Environment and Population Health, National Institute of Environmental Health, Chinese Center for Disease Control and Prevention, Beijing, 100021 China; 11 One Health Center of Excellence for Research and Training, School of Public Health, Sun Yat-Sen University, Guangzhou, China; 12 National Institute of Parasitic Diseases, Chinese Center for Disease Control and Prevention (Chinese Center for Tropical Diseases Research), Shanghai, China; 13 Integrated Research On Disaster Risk-IPO, Beijing, China; 14 State Key Lab of Earth Surface Processes and Resource Ecology, Beijing Normal University, Beijing, 100875 China; 15 Ministry of Education Key Laboratory for Earth System Modeling, and Department of Earth System Science, Tsinghua University, Beijing, China; 16 ASEAN Secretariat Indonesia; Integrated Research on Disaster Risk (IRDR), Jakarta, Indonesia; 17 Science and Innovation Department, World Meteorological Organization (WMO), 7 bis, Avenue de la Paix, BP2300, CH-1211 Geneva 2, Switzerland; 18 Max Planck Institute for Meteorology, Hamburg, Germany; 19 Chinese Center for Disease Control and Prevention, Beijing, China

**Keywords:** Climate change, One Health, International coordination and cooperation, Risk-oriented recommendations

## Abstract

It is unequivocal that human influence has warmed the planet, which is seriously affecting the planetary health including human health. Adapting climate change should not only be a slogan, but requires a united, holistic action and a paradigm shift from crisis response to an ambitious and integrated approach immediately. Recognizing the urgent needs to tackle the risk connection between climate change and One Health, the four key messages and recommendations that with the intent to guide further research and to promote international cooperation to achieve a more climate-resilient world are provided.

**Graphical Abstract:**

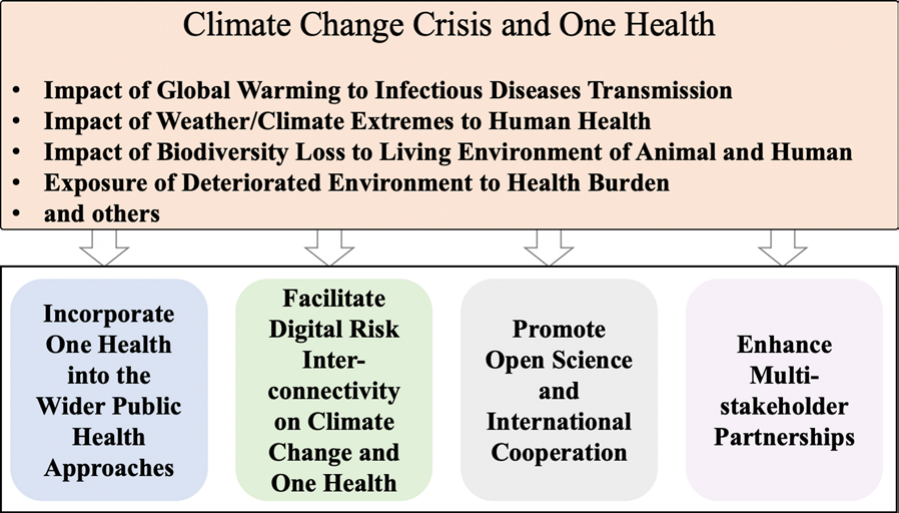

## Background

Climate change is one of the greatest threats to humankind in the twenty-first century [1]. It has been highlighted that there is an urgent need to investigate further the direct and indirect linkages of climate change with natural, biological, and other human-induced hazards. This will facilitate a better identification and understanding of cascading and complex hazards and risks in a systematic way to address climate change as a driver of hazards [2]. The shift towards a broader view and a more context-dependent definition of hazards requires a systematic approach to risk that considers hazard, vulnerability, exposure and capacity together and better understand their complex interaction.

Climate change is a global issue that goes beyond national borders. Tackling climate change requires intense international coordination and cooperation as recognized by the United Nations (UN) in 2015 when the Sendai Framework for Disaster Risk Reduction 2015–2030, the Sustainable Development Goals of Agenda 2030 and the Paris Agreement on Climate Change were adopted [3–5]. Ever since, more and more international organizations and partners are taking crosscutting actions and services to tackle climate change threat and disaster risk reduction for sustainable development. For example, the UN-wide initiative, Global Framework for Climate Services, led by World Meteorological Organization with World Health Organization (WHO), World Bank, the United Nations Development Programme, the International Federation of Red Cross and Red Crescent Societies, United Nations International Strategy for Disaster Reduction, World Food Programm, United Nations Educational, Scientific and Cultural Organization, Food and Agriculture Organization of the United Nations (FAO), United Nations Environment Programme (UNEP) directly involved, publishes reports annually on the state of climate services. Such reports give a view to “facilitating the development and application of methodologies for assessing adaptation needs”, especially for risk information and early warning systems [6].

While slowing down climate change is a priority, adaptation to the changing climate and trying to do our best to protect humans, animals and our shared environments from its negative impacts are an urgent need. It is clear that human health is intimately related to the health of our surrounding environments, including animals and plants and to the ecosystems on which all of us depend. The One Health approach is considered to be one of the best solutions to achieve optimal health and well-being outcomes [7]. A One Health High Level Expert Panel (OHHLEP) has been set up by four international organizations (FAO, World Organisation for Animal Health, UNEP and WHO) to collect, distribute and publicize reliable scientific information on the links between human, animal and environmental health [8].

Understanding the nature of hazards and the interconnected systemic risks is the basis for managing complex and cascading hazards and risks for better prediction, preparation and adaptation. This requires significant advances in the understanding of the role of anthropogenic systems, in recognition of the precursor signals and the associated correlations [9]. The interconnectivity of climate change, biodiversity, environmental pollution, wildlife habitats and human health requires that all of these issues be addressed as a whole, with their multi-way interactions. Protecting and restoring ecosystems, as well as making good use of its finite resources, are the key basis of human prosperity and well-being [10]. Recognizing this, the global community must garner a better understanding of the connection between climate change and One Health. In this short article, we stress the linkage between climate change and One Health, and propose recommendations for approaches and implementations from the One Health concept to action by taking the climate change into account. Here we present four key messages and recommendations that with the intent to guide further research and to promote international cooperation to achieve a more climate-resilient world.

## Incorporate One Health into the wider public health approaches

About 75% of all new infectious diseases have their origin in animals [11]. The coronavirus disease 2019 pandemic highlights the importance of holistic approach to prevent, detect and respond to health threats. From a One Health perspective, climate change can have considerable impact on factors that may affect disease transmission such as spatial distribution of vectors, encroachment into animal habitats, disruption of animal migration pathways and altered behavior and management by communities of animals, crops and their environments [12, 13]. Increased extreme weather/climate events in the context of climate change can exacerbate the disturbance on the environmental systems and deteriorate the situation [14]. Globalization increase the probability of cross-border and inter-species transmission of pathogens [15]. As pathogens can travel without passport and visa, governance of (pan) epidemic pathogens requires strengthen collaborations and information sharing between districts, nations and regions.

The public health, livestock, and agriculture sectors, etc., often adopt a ‘crisis response’ model for the prevention and control of emerging infectious diseases, waiting for an outbreak and then taking action. To deal with emerging infectious diseases, there should be a shift in traditional thoughts from crisis response to effective prevention. Human, animal and environmental health should be addressed as a whole, and interdisciplinary collaboration and communication across human-animal-environmental health should be developed.

It is vital to enhance surveillance of wild life reservoirs and investigation of potential pathogens (human, animal and plants), their environments and human interfaces in order to support early detection, prevention and control of emerging epidemics. It is necessary to set up a detecting system for professional population which has close contact with animals for early detection and warning of possible infection. Currently little action has been taken to prevent low-probability One Health related events (such as rare diseases), yet the distribution and development trends of diseases are projected to change significantly in the context of climate change. Therefore, future investigations should also focus on possible emerging health risks and their associated risks. Further research on the health impact of climate change should integrate a One Health approach with development of multi-modal environmental perception measurements and corresponding comprehensive monitoring technology and devices. One Health environmental indicators should be developed in order to achieve early monitoring and prediction as well as early warning of the synergistic effects of changes in climate and environment on integrated health.

## Facilitate digital risk interconnectivity on climate change and One Health

The characteristics of risks caused by climate change are cascading and compounding. The health impact of air pollutants, such as particulate matters (PM_2.5_ in particular), O_3_ and NOx have been widely investigated, yet most studies focus on all-cause mortality or respiratory and cardio-cerebrovascular diseases, with little focus on wider One Health impacts. Evidence suggested that climate change would exert influence on air quality through producing weather conditions which facilitate air pollution events [16]. Despite the fact that links between climate change, air pollution and health have been noted, the systemic risks caused by them and their interactions remain unclear. Therefore, it is very important to strengthen research on the understanding of mutual relations among climate change, air quality and health.

An operational system for chemical weather and chemical climate should be established to monitor and predict quantitatively chemical species in atmosphere and assess their impact on health. An all-cause assessment on health impact of climate change and environmental conditions is highly recommended to guide risk governance. Beside the physical and chemical changes in the Earth system, the role of human behavior and social characteristics should also be included in the assessment, as they are the key drivers of climate change and air pollution. The all-cause assessment requires evidences of connections between climate change, weather/climate extremes, environmental conditions, human behavior and social characteristics, animal and human health. This requires strong data support from various sectors, especially disease control departments, hospitals, administrations for food security and animal health, agencies in charge of environmental and meteorology. International Organizations could take leading role to facilitate such actions at national level, with specific standards, guidance, tools, checklists, and etc.

It is recommended that with the advances of new technologies such as digital earth tools and the data obtained, explicitly understanding of the complex earth system can be achieved undoubtedly. Extreme weather/climate forecasting can also benefit from the advancement in the digital earth tools. Development of digital tools to monitor energy use and greenhouse gas emission is of great importance for climate change mitigation. The spatial differences in terms of climate change impacts are critically important, and high-resolution analysis, evaluation and adaptation strategies for local regions should be identified as a key priority. It is believed that risk interconnectivity research on climate change, environment and health can significantly facilitate the development of more effective global assessments. Relevant prediction/projection systems will facilitate a pathway towards a better climate-resilience world. In support of these goals, comprehensive data observation, data standardization, earth system model development, big data and artificial intelligence application to bridge nature and social sciences should receive more attention. Furthermore, increased investments from all relevant sources (e.g., governmental sectors, non-governmental organizations, enterprises, agencies such as World Bank, etc.) are called for to promote interdisciplinary, action-orientated and mission-orientated research and integrated innovation in the corresponding fields. Both national and international projects are needed for tackling the risk interconnection and enhancing capacity of early detection, early identification, early warning and early response.

## Promote open science and international cooperation

“Open Science” and international cooperation are key for promoting the understanding of climate change impacts and better health governance. The “Open Science” principle can be a core game changer for bridging science, technology and innovation gaps between and within countries [17], and for driving science in service to society. An international network/framework, and/or programme/project with focus on climate change and One Health is proposed*.* This network/programme could promote international cooperation for better data and knowledge sharing with blocks of data acquisition, relational database, analysis/diagnosis tools, prediction/early warning techniques and science-based response system, based on the principles of “3Co” (Co-design, Co-produce and Co-deliver) [18].

Data sharing is regarded as the first priority for such cooperation. Therefore, establishment of national/international data centers that are integrated, open, shared and actively accessible are strongly recommended. Envisioning an inclusive world in the era of the transformation should be an important principle. Providing support to countries with limited capacity of evaluating and detecting the risk of climate change has reached a consensus to make sure no one is left behind. In addition, a call to promote scientific sharing of relevant knowledge, possibly via a knowledge exchange or hub, to raise awareness of the threats of climate and other environmental changes and what actions work to build a climate resilient, low-emission and sustainable society.

## Enhance multi-stakeholder partnerships

Combating climate change is not only a scientific issue, but also requires multi-stakeholder participation. Multi-sectoral cooperation and new ways of science-based governance are highly recommended. The establishment of several networks/platforms at national/international levels to enhance multi-stakeholder partnerships is called for continued advancement in climate change and integrated health governance. Climate Change and One Health committee at national level is proposed in order to promote science-based governance and multi-sector collaboration. Such committee can coordinate universities, scientific research institutions and government agencies in disease control, medical treatment, climate change adaptation, agriculture and environmental protection by adopting the latest research results into social practice and governance processes. The United Kingdom (UK) provides a very good example on infectious disease control through multi-stakeholder collaboration. A multi-agency cross-government ‘Human Animal Infections and Risk Surveillance (HAIRS) group’ was set up in 2004, which brought together representatives from human and animal health from the Departments and Agencies across the UK, to identify emerging zoonoses which may pose a future threat [19].

Developing a collaborative network with actionable goals, such as One Health association with government agencies, academic institutes, and enterprises is called for to facilitate multi-stakeholder participation. Establishment of an international research and education alliance is recommended to facilitate multi-disciplinary collaboration between universities and scientific research institutions, for addressing the relevant global governance needs. The major objectives of the alliance are to incorporate climate change and One Health into the general curriculum and postgraduate training directions of higher education, and develop qualified interdisciplinary scientific experts to achieve the broad goal of a health planet and healthy human societies.

## Conclusions

The warmed earth climate is seriously affecting the planetary health including human health [14]. A One Health approach is required to tackle climate change by implementing a united, holistic action and a shift from crisis response to prevention.

**Fig. 1:**
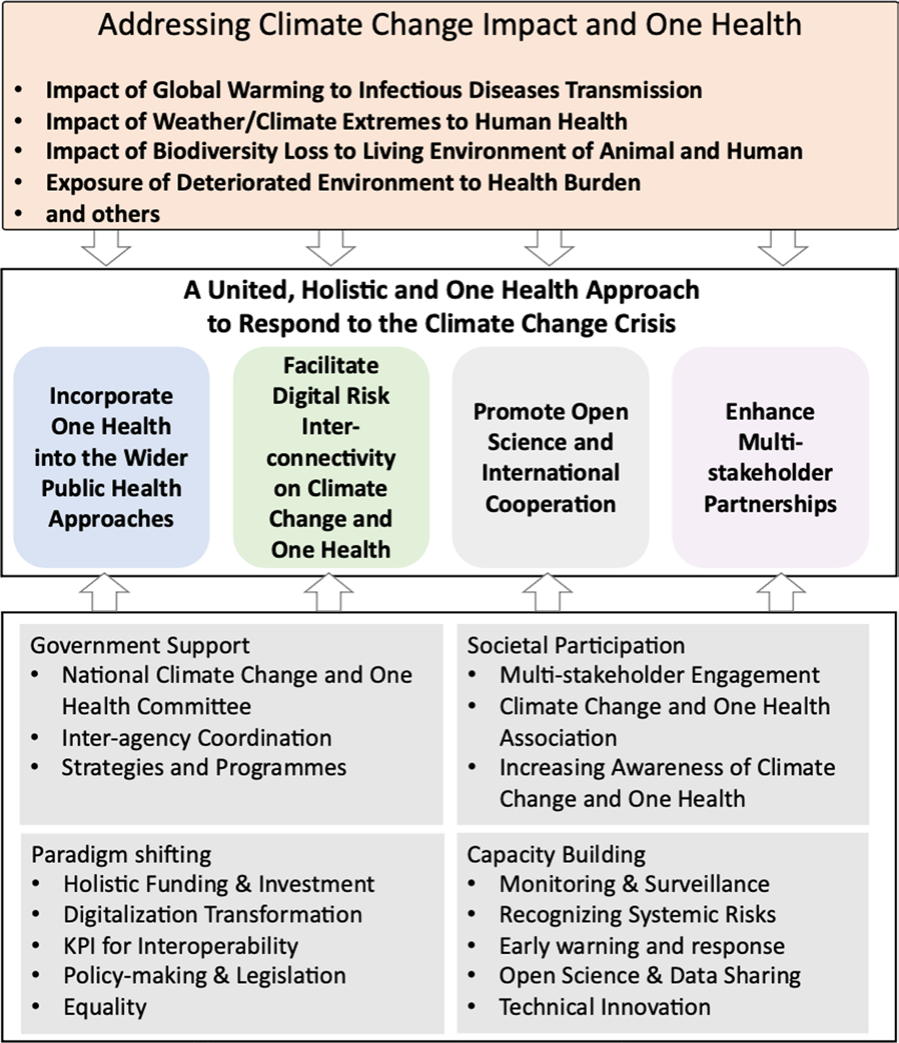
Recommendations for tackling climate change crisis and one health

It is recommended to establish a Climate Change and One Health network at the national level so as to promote science-based governance and multi-sector collaboration and multi-stakeholder partnership. Regional alliances and cooperation is required to surveil and investigate wild life reservoirs of potential pathogens (human, animal and plants), their environments and human interfaces, as well as possible emerging diseases and health risks. Supported by national and regional networks and alliances, the establishment of a Climate Change and One Health intergovernmental steering committee is proposed to tackle the integrated and systemic issues. Climate change governance would further benefit from the explicit understanding of the complex interconnectivity on climate change and One Health through an all-cause assessment and the establishment of integrated and open bigdata center gathering all relevant data and baselines for early warning and effective response. To illustrate the recommendations for tackling climate change crisis and One Health, a diagram is given in Fig. 1 to summarize the main viewpoints we proposed.

It should be noted that the implementation of the above recommendations could be quite challenging. The existed old and rigid public health structure with well-defined responsibilities and boundaries are expected to be changed. The limited inter-cooperability between multi-sectors due to non-unified data recording and storage standards have to adapt to the requirement of uniformed formats. The limited resources and funding available to allow multi-stakeholder collaboration should be expanded. In spite of these challenges, in a climate-emergency era climate change threats have become a top priority for the governments around the world, as reflected by the enthusiastic participation and commitment by nearly 200 countries to the COP-26 pact in Glasgow, UK held last year. We believe these challenges could be solved with strong support from the governments, scientific communities, enterprises and the public in their rapid actions to mitigate climate change threats.

## Data Availability

All the materials used in this manuscript can be accessed through the DOI or website listed in References.
